# COVID-19 Vaccine Hesitancy among New Jersey Teachers and Impacts of Vaccination Information Dissemination

**DOI:** 10.3390/vaccines11020466

**Published:** 2023-02-17

**Authors:** Kimberly T. Nguyen, Juhi Aggarwal, Maryanne L. Campbell, Stephanie Shiau, Derek G. Shendell

**Affiliations:** 1 NJ Safe Schools Program, Rutgers School of Public Health (SPH), Rutgers University, Piscataway, NJ 08854, USA; 2 Department of Epidemiology & Biostatistics, Rutgers School of Public Health (SPH), Piscataway, NJ 08854, USA; 3 Department of Environmental & Occupational Health & Justice, Rutgers School of Public Health (SPH), Piscataway, NJ 08854, USA

**Keywords:** COVID-19, New Jersey, safety and health, teachers, vaccine education, vaccine hesitancy, immunizations

## Abstract

Vaccine hesitancy continues to be prevalent in the United States, especially in relation to the COVID-19 vaccines and its boosters, which have been made increasingly available for public use as the pandemic has progressed. There continues to be concern surrounding the safety and health of secondary or high school education professionals as they transition back to in-person learning and working opportunities. The present study highlights how information dissemination regarding the COVID-19 vaccine has varied among New Jersey secondary or high school teachers throughout the pandemic. The survey was completed online through the PsychData platform by 269 participants between March and July 2022. Participants received the opportunity to complete the survey via email. Afterwards, data were exported and analyzed using Microsoft Excel and SAS 9.4 Analytics Software and stratified by various clinical and demographic-based variables. While trusted agencies and media outlets identified by participants varied, most participants identified the Centers for Disease Control and Prevention (65.4%), primary care providers (37.5%), and state health departments (28.6%) as their top trusted sources for information related to COVID-19 vaccines. Overall, COVID-19 vaccination advocacy and educational efforts should continue across the state of New Jersey and elsewhere, especially as more variants emerge and boosters become available.

## 1. Introduction

The prevalence of vaccine hesitancy has been a challenge for medical professionals and public health advocates alike for several decades, even outside the context of coronavirus disease 2019 (COVID-19) caused by severe acute respiratory syndrome coronavirus 2 (SARS-CoV-2) [1]. Vaccine hesitancy can be defined as either the delay in the acceptance of or the rejection of vaccine administration as a result of propaganda put forth by anti-vaccine groups and continues to be a persistent problem among many populations [2]. Vaccine hesitancy increases as populations focus more of their attention on the false accusations and adverse reactions, as opposed to the benefits, associated with vaccinations [2]. High rates of vaccine hesitancy have been reported within the general population and continue to be prevalent amongst influential figures, such as healthcare professionals and school educators [3,4]. Given the impact these figures have on the community, it is important to understand and challenge the reasons behind vaccine hesitancy throughout the pandemic.

While a variety of vaccinations are mandatory for school entry in the United States, exemptions can be granted on a case-by-case basis [2]. School education professionals are at an increased risk of contracting preventable diseases due to close contact with school-aged children who are often subjected to receiving parental consent prior to vaccine administration [5]. During the COVID-19 pandemic, to protect the health and safety of school education professionals while working face-to-face, New Jersey (NJ) implemented a statewide policy, effective 18 October 2021, that required educators to either receive the vaccine, or undergo regular COVID-19 testing if they chose to remain unvaccinated, before returning to the workplace [6]. Further, beginning 15 August 2022, the policy no longer required unvaccinated educators to undergo routine COVID-19 testing [7]. With over 75.0% of NJ’s overall population presently fully vaccinated against COVID-19, a portion of the population remains unprotected [8]. Exploring the impact of vaccine hesitancy among adults and school-age children will provide valuable insights on how mass vaccination efforts can be both improved and deemed successful, as well as inform future guidance to schools and districts as students and adult administrators, teachers, and educational support professionals transition back to full-time in-person schooling.

Across vaccines with emergency use authorization (EUA), the COVID-19 vaccine manufactured by Pfizer-BioNTech was the first ever to be approved for use in children and adolescents in the United States (U.S.). On 11 December 2020, the U.S. Food and Drug Administration (FDA) authorized the EUA of the Pfizer-BioNTech COVID-19 vaccine for the prevention of COVID-19 SARS-CoV-2 for individuals 16 years of age and older. Five months later, this EUA extended eligibility to include adolescents 12 to 15 years of age [9]. This EUA was then further extended to include children between 5 to 11 years of age on 29 October 2021 [10], providing coverage for all the ages of our target population, K-12 schools.

The vaccine plays a crucial role in the fight against COVID-19. According to the U.S. Centers for Disease Control and Prevention (CDC), the initial use of the approved vaccines was reported to be approximately 90.0% effective in protecting patients against symptomatic infection, severe illness, and death [11]. Furthermore, according to the FDA, the Pfizer-BioNTech vaccine was similarly found to initially be 90.7% effective in preventing COVID-19 in children who were 5 through 11 years of age [11]. In addition, researchers have repeatedly reported how hospitalization rates related to COVID-19 are higher amongst unvaccinated patients, regardless of the child’s age [10,12,13,14].

Despite studies and statistics available to date, there has been a substantial number of parents who are advocating against receiving the vaccine for both themselves and their children [15]. Parents often rely on a variety of sources when making decisions regarding the safety and well-being of their children, making the validity of those sources important. In a recent study, researchers reported most parents turn to their child’s physician as opposed to the television and internet when seeking vaccination information [16,17]. Parents also report difficulty understanding how an EUA of a vaccine impacts vaccine safety, efficacy, and side effects [16]. Many parents harbor concerns regarding the quickness of vaccine production and limited information available regarding the long-term effects of receiving the vaccine [18]. With an abundance of contradicting news sources and constant fluctuation in the proposed preventative safety guidelines from public health agency officials (e.g., CDC), many parents, including teachers, have been left confused and hesitant when it comes to vaccinating themselves and their children.

Researchers have reported some correlations between different demographic factors and COVID-19 vaccine hesitancy as well. Key findings from a recent survey noted rural, lower income, uninsured individuals and larger households were less likely to get vaccinated [19]. Additionally, adult women were less likely to report intentions of getting themselves vaccinated than men [19]. Another survey conducted in an urban setting concluded COVID-19 vaccine hesitancy was higher among Non-Hispanic Black adults versus Non-Hispanic White adults [19]. Another study found higher rates of COVID-19 vaccine hesitancy among parents of publicly insured children compared with privately insured children [20]. Given the severity of COVID-19, researchers, physicians, and public health officials must continue to dispel misconceptions regarding the COVID-19 vaccine and encourage vaccine intake, especially amongst high-risk populations.

The present study aims to describe how information dissemination regarding the COVID-19 vaccine has varied among NJ secondary or high school (HS) teachers throughout the pandemic. Previous recent research studies have identified that teacher perceptions, beliefs, and attitudes towards the COVID-19 vaccines are crucial determinants of vaccine hesitancy [21,22]. Therefore, findings of this study and others worldwide will become increasingly relevant in the coming years, as more life-threatening diseases come about, and public health officials and healthcare providers alike need to identify how to better advocate to the general population how these new vaccines are necessary to prevent serious illness. The population used in this study is of interest given the work and influence their decisions regarding safety and health have on students and their communities. Additionally, given that teachers are often required to interact with their students and colleagues on a face-to-face basis, it is important they remain well informed on the best practices to protect their health against COVID-19 and other infectious illnesses.

## 2. Materials and Methods

From 2014 to 2022, the New Jersey Safe Schools (NJSS) program provided safety and health (S&H) trainings to NJ teachers seeking to be endorsed for work-based learning (WBL) (formerly called school-sponsored structured learning experiences in NJ) in one or more career clusters in career and technical education (CTE). During these eight school years, over a thousand teachers completed the set of code required by NJSS trainings [23]. After removing inactive email addresses (meaning the teacher no longer works at the school in which they were employed when they registered/took the trainings with NJSS), a total of 1015 participants were eligible for this online survey, which was emailed to current and previous training participants. Participants were able to complete the survey on their own time using their own personal electronic devices in the environment of their choosing on the PsychData platform. To be included in this study, participants had to have previously completed the WBL trainings and start the online survey provided. Participants were excluded from this study if they did not start or completed only 1–2 questions of the online survey provided. A total of 269 of 1015 participants (26.5%) completed or partially completed the survey. This survey was distributed in two rounds (March–April 2022 and June–July 2022). Results were combined for this study’s analyses.

The present study’s survey included 28 multi-part questions about vaccine status information, experience with the COVID-19 vaccine, vaccine perceptions and opinions, COVID-19 education information, COVID-19 safety practices, COVID-19 case information, and demographics.

Overall, the data were stratified by vaccination status at the time of survey, number of doses of vaccine at the time of survey, booster status at the time of survey, age, teaching experience (both overall and in NJ), race/ethnicity, county of work, and gender. County was stratified by Cumberland and Ocean counties and other counties. Cumberland County was of interest because it had the lowest rate of adults receiving the influenza vaccine in NJ, at 28.6% in 2020 [24]. Ocean County was of interest because it was the origin of the 2019 Measles outbreak in NJ [25]. As mentioned in previous studies, age, gender, and race/ethnicity are demographic variables of interest and have been associated with an increase in vaccine hesitancy [3]. Survey questions were taken and adapted from the U.S. CDC Behavioral Risk Factor Surveillance Survey, the U.S. CDC Vaccine Confidence Survey Question Bank, and previous surveys conducted by NJSS [26,27,28,29,30]. Overall vaccine hesitancy is measured by vaccine status and booster status. Those who do not get vaccinated are interpreted as vaccine hesitant.

Data were analyzed through Microsoft Excel (Version 2208 Build 16.0.15601.20446) and SAS Analytics Software 9.4 (Cary, NC). Fisher’s Exact tests were conducted to compare survey results across various stratifications. Calculated *p*-values below 0.05 were considered statistically significant. This study was approved by the Institutional Review Board (IRB or Ethics Committee) of Rutgers, the State University of New Jersey (IRB protocol code: 2022000237).

## 3. Results and Discussion

### 3.1. Demographics

This study’s sample population of CTE teachers supervising secondary or HS students in WBL spans over the three regions of NJ, where 43.7% of participants were from North NJ, 36.7% from Central NJ, and 19.7% from South NJ. A majority of participants (76.4%) self-identified as Non-Hispanic White (NHW), and 23.6% were categorized as “other”. Other consisted of people who self-identified as American Indian or Alaskan Native, Middle Eastern or North African, Hispanic Asian, Hispanic Black, Hispanic White, Non-Hispanic Asian, Non-Hispanic Black, Multiracial, or the option other with an ability to write in an answer. A total of 64.6% self-identified as female, 31.4% identified as male, and 1.3% identified as other or multigender. The average birth year for the teachers was 1976 (SD 9.6), making the average age 46. The average number of years teaching overall was 16.2 (SD 7.4). The majority (63.8%) had a master’s degree and 2.6% had a doctoral degree. Teachers had an average of 6.4 (SD 2.6) years of post-secondary education (Supplemental Table S1).

### 3.2. COVID-19 Education Information—Identifying Sources

Throughout the COVID-19 pandemic, citizens across the country relied on a variety of sources to obtain the most up-to-date information regarding safety and preventative protocols when making informed decisions about their health. Most survey participants reported they knew where to obtain accurate information about the COVID-19 vaccines (91.4% overall—Table 1). As the pandemic progressed, the number of trusted sources of COVID-19 content grew. This suggested that individuals had more choices regarding information outlets. Valued sources of information included U.S. CDC, U.S. FDA, employers/friends/family, health insurers, hospital system websites, local health officials, news sources, nurses, pharmacists, primary care physicians, professional organizations, religious leaders, state health departments, online publishers of medical information, social media, and union leaders. For these questions, prior to May 2022, the answer choices were arranged such that “employer, family, and friends/Food and Drug Administration (FDA)“ were combined and listed as one single selection option. Following May 2022, participants were given the opportunity to select “Food and Drug Administration (FDA)” and “employer, family, and friends” separately. For the descriptive analysis, participants who completed the survey prior to this change and selected the original answer choice were counted as both “Food and Drug Administration (FDA)” and “employer, family, and friends”.

Given the novelty and constant evolution of the COVID-19 virus, accurate sources of information were essential to those wishing to make informed decisions regarding their health and safety. They were asked to identify their top three most trusted sources from the source selection mentioned. While most participants selected three different sources when answering this question (54.9%), there were some participants who selected more (24.5%). They were also given the opportunity to submit other sources not already identified. Overall, the CDC, primary care physicians, and state health departments were identified as the participants’ top three most trusted sources of information about the COVID-19 vaccine (75.5%, 43.3%, and 33.3%, respectively—Table 1).

This study asked teachers to identify three of their most trusted sources regarding the COVID-19 vaccine. Overall, the general study population chose the CDC, FDA, and local health departments most frequently. Different demographics were associated with different sources of information.

When stratified by gender, race, counties, number of vaccine doses received, and COVID-19 diagnosis status, all groups identified the CDC as one of their top three sources for COVID-19 information, followed by primary care providers and state health departments (Table 2). There was a borderline statistical difference between participants who selected the CDC when stratifying by vaccine dose status (*p* = 0.08). There was a difference between participants who did and did not receive the booster who selected primary care providers (*p* = 0.03). There was a borderline statistical difference between participants who did and did not receive the booster who selected the CDC (*p* = 0.07).

When stratified by vaccination status, most of both the vaccinated and unvaccinated participants identified the CDC as one of their top three sources for COVID-19 information (79.1% vs. 33.3%, respectively) (Table 2). Vaccinated individuals identified primary care providers (45.6%) and state health departments (35.8%) as their second and third sources for COVID-19 information (Table 2). Unvaccinated individuals identified employers, family, and friends (22.2%) and the FDA/primary care providers (16.7%) as their second and tied-third sources for COVID-19 information (Table 2). There was a difference between vaccinated and unvaccinated participants who selected the CDC (*p* ≤ 0.001), primary care providers (*p* = 0.02), and state health departments (*p* = 0.001).

### 3.3. COVID-19 Education Information

Researchers have identified specific health conditions believed to impact the severity of and the susceptibility of patients to the COVID-19 virus: cancer, immunocompromised state due to therapy or disease, obesity, diabetes, cardiovascular disease, pulmonary disease, and rheumatological conditions. When the COVID-19 vaccine was first released, priority administration was given to special employment populations (healthcare workers, educators, etc.) as well as patients suffering from these seven conditions. Overall, obesity was the top reported condition among participants who responded to this question (14.2–15.0%) (Supplemental Table S2).

When stratified by gender, the top three comorbidities reported among females were obesity, cardiovascular disease, and immunocompromised states due to therapy or disease (Supplemental Table S2). Meanwhile, the top three comorbidities reported among males were obesity, diabetes, and immunocompromised states due to therapy or disease (Supplemental Table S2). There was a difference between participants who selected diabetes for this question (*p* ≤ 0.001).

When stratified by vaccination status, the top three comorbidities among participants who were vaccinated were obesity, immunocompromised state due to therapy or disease, and diabetes/cardiovascular disease (Supplemental Table S2). Meanwhile, the top three comorbidities reported among participants who were unvaccinated were obesity, diabetes, and pulmonary disease (Supplemental Table S2).

When stratified by booster status, the top three comorbidities among participants who received the booster were obesity, diabetes, and immunocompromised state due to therapy or disease/cardiovascular disease (Supplemental Table S2). Meanwhile, the top three comorbidities reported among participants who did not receive the booster were obesity, immunocompromised state due to therapy or disease, and cardiovascular disease (Supplemental Table S2).

When stratified by COVID-19 diagnosis status, the top three comorbidities among participants who have received a positive diagnosis were diabetes, cardiovascular disease, and immunocompromised state due to therapy or disease (Supplemental Table S3). Meanwhile, the top three comorbidities among participants who received a positive diagnosis were obesity, diabetes, and immunocompromised states due to therapy or disease (Supplemental Table S2). There was a difference between participants who selected cardiovascular disease for this question (*p* = 0.05).

After the COVID-19 vaccine was released to the public, the recommendation to receive it from others also influenced whether widespread administration occurred. Survey participants were asked to state the likelihood that they would recommend getting the COVID-19 vaccine, with the majority (76.0–80.9%) identifying that they would be at least somewhat likely to do so (Supplemental Table S2).

When stratified by all factors, most participants reported that they would be at least somewhat likely to recommend the vaccine (Supplemental Table S2). There was a difference between vaccinated and unvaccinated participants for this question (*p* ≤ 0.001). There was also a difference between participants who did and did not receive the booster (*p* ≤ 0.001), as well as between participants who did and did not receive a positive COVID-19 diagnosis for this question (*p* ≤ 0.001).

### 3.4. COVID-19 Safety Practices Information—Social Distancing

Further, throughout the pandemic, both before and after the availability of the COVID-19 vaccine, the use of social distancing practices was encouraged both in public and private spaces to prevent the spread of disease. The survey provided asked participants to identify the likeliness they would maintain at least a six-foot distance from others in public spaces, private gatherings, and workplaces. Overall, most participants reported they would be at least somewhat likely to follow these social distancing protocols in public spaces (57.5–57.8%) (Supplemental Table S3). However, overall, most participants reported it was not at all likely they would follow social distancing protocols while at small private gatherings and working with people who they do not live with (52.0–53.5% vs. 53.3–54.0%, respectively) (Supplemental Table S3).

When stratified by gender, race, and counties, most participants (57.5–57.7%) reported they would be at least somewhat likely to maintain a six-foot distance from others who do not live in their home while in public spaces (Supplemental Table S3). There was also a difference between these two racial/ethnicity groups (*p* = 0.04).

When stratified by race, NHW were more likely to report it was not at all likely they would following social distancing protocols in this work setting when compared to other racial/ethnicity groups (56.0% vs. 27.3%, respectively) (*p* = 0.03) (Supplemental Table S3).

When stratified by vaccine status, vaccinated participants were more likely to be at least somewhat likely to practice maintaining a six-foot distance from others who do not live in their home while in public spaces when compared to unvaccinated participants (58.6% vs. 44.4%, respectively) (Supplemental Table S3). Both participants who were vaccinated and unvaccinated reported it was not at all likely they would maintain at least a six-foot distance from people who do not live in their home while at small private gatherings (51.2% vs. 66.7%, respectively) (Supplemental Table S3). Most of both participants who were vaccinated and unvaccinated reported it was not at all likely they would follow social distancing protocols in the work setting (53.0% vs. 55.6%, respectively) (Supplemental Table S3). However, there were no statistical differences in these cases.

When stratified by vaccine dose status, participants who received two doses of the vaccine were more likely to at least somewhat maintain a six (6)-foot distance from others who do not live in their home while in public spaces when compared to participants who received one dose of the vaccine (60.2% vs. 35.7%, respectively) (Supplemental Table S3).

When stratified by vaccine dose status, both participants who received one dose and two doses of the vaccine reported it was not at likely they would remain at least six feet from people who do not live in their home while at small private gatherings (57.1% vs. 50.7%, respectively) (Supplemental Table S3).

When stratified by vaccine dose status, most of both participants who received one dose and two doses of the vaccine reported it was not at all likely they would follow social distancing protocols in the work setting (64.3% vs. 52.2%, respectively) (Supplemental Table S3).

When stratified by booster status, most of both participants who did and did not receive the booster reported they would be at least somewhat likely to maintain a six-foot distance from others who do not live in their home while in public spaces (64.7% vs. 37.5%, respectively) (*p* = 0.003) (Supplemental Table S3).

When stratified by booster status, participants who received the booster were more likely to maintain at least a six-foot distance from people who do not live in their home while at small private gatherings when compared to participants who did not receive the booster (52.7% vs. 31.3%, respectively) (Supplemental Table S3).

When stratified by booster status, participants who did not receive the booster were more likely to report it was not at all likely they would follow social distancing protocols in the work setting when compared to participants who did receive the booster (68.8% vs. 48.5%, respectively) (*p* = 0.02) (Supplemental Table S3).

### 3.5. COVID-19 Safety Practices Information—Masks and Facial Coverings Use

Public health officials established the use of masks/facial coverings to help limit the spread of disease during the height of the pandemic, leading many states and territories to mandate their use by citizens while in public settings. Participants were asked to identify the likeliness they would use a mask/facial covering while in a neighborhood, shopping inside a store, inside a friend’s house, and in other outdoor spaces. Overall, participants reported it was not at all likely they would wear a mask while in work settings outside the home (51.2–52.8%), going for a walk in their neighborhood (82.8–83.6%), inside a friend’s house (72.9–73.7%), or in other outdoor spaces (82.8–83.6%) (Supplemental Table S4). Overall, most participants reported they would be at least somewhat likely to wear a mask while using public transportation (64.8–66.7%) and while shopping inside a store (55.3–57.7) (Supplemental Table S4). 

When stratified by gender, males were more likely to report it was not at all likely they would wear a mask in the work setting outside the home when compared to females (58.3% vs. 49.7%, respectively) (*p* = 0.001) (Supplemental Table S4). In addition, most of both males and females reported they would be at least somewhat likely to wear a mask while using public transportation (68.7% vs. 59.8%, respectively) (Supplemental Table S4).

When stratified by race, most of both the participants who identified as Non-Hispanic Whites and other racial/ethnicity groups reported it was not at all likely they would wear a mask while going for a walk in their neighborhood (85.9% vs. 63.6%, respectively) (Supplemental Table S4), and how it was not at all likely they would wear a mask while inside a friend’s house (75.9% vs. 54.5%, respectively, *p* = 0.01 and *p* = 0.03, respectively) (Supplemental Table S4). 

When stratified by vaccination status, over half of both participants who were vaccinated and unvaccinated reported it was not at all likely they would wear a mask in the work setting outside the home (51.2% vs. 72.2%, respectively) (Supplemental Table S4). When stratified by vaccination status, participants who were vaccinated were more likely to report they would be at least somewhat likely to wear a mask while using public transportation when compared to participants who were unvaccinated (66.0% vs. 50.0%, respectively) (Supplemental Table S4). When stratified by vaccination status, both participants who were and were not vaccinated reported it was not at all likely they would wear a mask while going for a walk in their neighborhood (82.8% vs. 88.9%, respectively) (Supplemental Table S4). When stratified by vaccination status, about three-quarters of both participants who were vaccinated and unvaccinated reported it was not at all likely they would wear a mask while shopping inside a store (73.0% vs. 72.2%, respectively) (Supplemental Table S4). When stratified by vaccination status, over half of both participants who were vaccinated and unvaccinated reported it was not at all likely they would wear a mask while inside a friend’s house (62.8% vs. 77.8%, respectively) (Supplemental Table S4).

When stratified by vaccine dose status, participants who received one dose of the vaccine were more likely to report it was not at all likely they would wear a mask in the work setting outside the home compared to participants who received two doses of the vaccine (71.4% vs. 49.8%, respectively) (Supplemental Table S4). When stratified by vaccine dose status, both participants who received one and two doses of the vaccine reported they would be at least somewhat likely to wear a mask while using public transportation (64.3% vs. 66.2%, respectively) (Supplemental Table S4). When stratified by vaccine dose status, over three-quarters of both participants who received one and two doses of the vaccine reported it was not at all likely they would wear a mask while going for a walk in their neighborhood (78.6% vs. 83.1%, respectively) (Supplemental Table S4). When stratified by vaccine dose status, participants who received one dose of the vaccine were more likely to report it was not at all likely they would wear a mask while shopping inside a store compared to participants who received two doses of the vaccine (64.3% vs. 40.3%, respectively) (Supplemental Table S4). When stratified by vaccine dose status, about three-quarters of both participants who received one dose and two doses of the vaccine reported it was not at all likely they would wear a mask while inside a friend’s house (78.6% vs. 72.6%, respectively) (Supplemental Table S4). When stratified by vaccine status, about three-quarters of both participants who were vaccinated and unvaccinated reported it was not at all likely they would wear a mask while in other outdoor spaces (79.5% vs. 83.3%, respectively) (Supplemental Table S4). When stratified by vaccine dose status, about three-quarters of both participants who received one dose and two doses of the vaccine reported it was not at all likely they would wear a mask while in other outdoor spaces (78.6% vs. 79.6%, respectively) (Supplemental Table S4).

When stratified by booster status, participants who did not receive the booster were more likely to report it was not at all likely they would wear a mask in the work setting outside the home compared to participants who did receive the booster (72.9% vs. 44.9% respectively, *p* = 0.003) (Supplemental Table S4). When stratified by booster status, participants who did not receive the booster were more likely to report it was not at all likely they would wear a mask while using public transportation compared to participants who did receive the booster (54.2% vs. 26.9%, respectively, *p* ≤ 0.001) (Supplemental Table S4). When stratified by booster status, over three-quarters of both participants who did and did not receive the booster reported it was not at all likely they would wear a mask while going for a walk in their neighborhood (82.6% vs. 83.3%, respectively) (Supplemental Table S4). When stratified by booster status, participants who received the booster were more likely report it was at least somewhat likely they would wear a mask while shopping inside a store when compared to those who did not receive the booster (61.0% vs. 45.9%, respectively) (Supplemental Table S4). When stratified by booster status, over half of both participants who did and did not receive the booster reported it was not at all likely they would wear a mask while inside a friend’s house (70.7% vs. 81.3%, respectively) (Supplemental Table S4). When stratified by booster status, over three-quarters of both participants who received one dose and two doses of the vaccine reported it was not at all likely they would wear a mask while in other outdoor settings (77.8% vs. 85.4%, respectively, *p* = 0.05) (Supplemental Table S4).

When stratified by COVID-19 diagnosis status, over half of both participants who have and have not received a positive diagnosis reported they would be at least somewhat likely to wear a mask while using public transportation (54.8% vs. 74.2%, respectively, *p* = 0.01) (Supplemental Table S4). 

### 3.6. COVID-19 Safety Practices Information—Concern of Contracting or Infection with SARS-CoV-2

Given the severity of the symptoms and risk of death associated with the COVID-19 virus, concern of contracting and/or spreading the disease was prevalent during all points of the pandemic. To better understand this, participants were asked to identify their level of concern regarding contracting COVID-19 both inside and outside the workplace, as well as the risk of infecting friends and family at any point. Overall, most participants reported they were at least a little concerned about contracting COVID-19 while at work (74.2–78.1%) (Supplemental Table S5). Overall, most participants reported they were at least a little concerned about contracting COVID-19 while outside of work (63.5–66.6%) (Supplemental Table S5). Overall, most participants reported they were at least a little concerned about infecting their friends and family with COVID-19 (77.7–81.4%) (Supplemental Table S5). Those participants who were older tended to be most concerned with contracting COVID-19 outside of work as compared to their younger colleagues.

When stratified by race, about three-quarters of both the participants who identified as NHW and other racial/ethnicity groups reported they were at least a little concerned about contracting COVID-19 while at work (73.8% vs. 86.4%, respectively), and about contracting COVID-19 while outside of work (62.3% vs. 81.8%, respectively) (Supplemental Table S5). There was also a difference between the responses of these two racial/ethnicity groups (*p* = 0.002 and *p* = 0.08, respectively). When stratified by vaccination status, participants who were vaccinated were more likely to be at least a little concerned about contracting COVID-19 while at work (78.1% vs. 27.8%, respectively, *p* ≤ 0.001) and while outside of work (66.5% vs. 27.8%, respectively, *p* = 0.02), and about infecting their friends and family with COVID-19 (81.4% vs. 33.3%, respectively, *p* ≤ 0.001) (Supplemental Table S5). Overall, those who were more worried were more likely to get the COVID-19 vaccine.

When stratified by vaccine dose status, over three-quarters of both participants who received one dose or two doses of the vaccine reported they were at least a little concerned about contracting COVID-19 while at work (78.6% vs. 78.1%, respectively) (Supplemental Table S5). When stratified by vaccine dose status, over three-fifths of both participants who received one dose or two doses of the vaccine reported they were at least a little concerned about contracting COVID-19 while out of work (71.4% vs. 66.2%, respectively) (Supplemental Table S5). When stratified by vaccine dose status, over four-fifths of both participants who received one dose or two doses of the vaccine reported they were at least a little concerned about infecting their friends and family with COVID-19 (85.7% vs. 81.1%, respectively) (Supplemental Table S5).

When stratified by booster status, over three-fifths of both participants who received and did not receive the booster reported they were at least a little concerned about contracting COVID-19 while at work, though more among participants receiving the booster (83.2% vs. 60.4%, respectively) (Supplemental Table S5). When stratified by booster status, participants who received the booster were more likely to report they were at least a little concerned about contracting COVID-19 while outside of work when compared to those who did not receive the booster (71.9% vs. 47.9%, respectively, *p* = 0.01) (Supplemental Table S5). When stratified by booster status, over three-quarters of participants who both received and did not receive the booster reported they were at least a little concerned about infecting their friends and family with COVID-19 (83.2% vs. 75.0%, respectively) (Supplemental Table S5).

When stratified by COVID-19 diagnosis, over half of both participants who did or did not receive a positive diagnosis reported they were at least a little concerned about contracting COVID-19 while outside of work (52.8% vs. 74.2%, respectively) (Supplemental Table S5). There was also a difference between the responses of these two diagnosis groups (*p* ≤ 0.001). When stratified by COVID-19 diagnosis, over three-quarters of both participants who did or did not receive a positive diagnosis reported they were at least a little concerned about contracting COVID-19 while outside of work (77.4% vs. 79.0%, respectively) (Supplemental Table S5).

### 3.7. Comparison of Responses by Safety Question

When assessing the participants’ likelihood of wearing a mask and maintaining at least a six-foot distance from others at work, most participants who reported it was not at all likely they would wear a mask while in a work setting outside the home also reported it was not at all likely they would maintain at least a six-foot distance from people at work (37.8%). About 5% of participants who reported it was extremely likely they would wear a mask in a work setting outside the home also reported it was extremely likely they would maintain at least a six-foot distance from people at work. About 14% of participants who reported it was extremely likely they would wear a mask in a work setting outside the home also reported it was somewhat likely they would maintain at least a six-foot distance from people at work (Supplemental Table S8).

When assessing the participants’ likelihood of wearing a mask and maintaining at least a six-foot distance from others while in indoor places with friends and family, most participants who reported it was not at all likely they would wear a mask while inside a friend’s house also reported it was not at all likely they would maintain at least a six-foot distance from others while at small gatherings (43.7%). About 2% of participants who reported it was extremely likely they would wear a mask while inside a friend’s house also reported it was extremely likely they would maintain at least a six-foot distance from others while at small gatherings. About 17% of participants reported it would be at least somewhat likely they would both wear a mask while inside a friend’s house and maintain at least a six-foot distance from others while in indoor places with friends and family (Supplemental Table S9).

When assessing the participants’ likelihood of wearing a mask while on public transportation and maintaining at least a six-foot distance from others when surrounded by people who do not live in their homes, most participants who reported it was not at all likely they would wear a mask while on public transportation also reported it was not at all likely they would maintain at least a six-foot distance from people who do not live in their home (21.2%). About 44% of participants reported it would be at least somewhat likely they would both wear a mask while on public transportation and maintain at least a six-foot distance from people who do not live in their home (Supplemental Table S10).

When assessing the participants’ likelihood of wearing a mask while in a store and maintaining at least a six-foot distance from others when surrounded by people who do not live in their homes, most participants who reported it was not at all likely they would wear a mask while at a store also reported it was not at all likely they would maintain at least a six-foot distance from people who do not live in their home (29.7%). About 43% of participants reported it would be at least somewhat likely they would both wear a mask while at a store and maintain at least a six-foot distance from people who do not live in their home (Supplemental Table S11).

When assessing the participants’ concern of contracting COVID-19 while at work and the likelihood they would wear a mask while in work settings outside the home, most participants who reported they were not at all concerned about contracting COVID-19 at work also reported it was not at all likely they would wear a mask in a work setting outside the home (22.1%). About 27% of participants reported they were at least somewhat concerned/would be somewhat likely they would both contract COVID-19 at work and wear a mask in a work setting outside the home (Supplemental Table S12).

When assessing the participants’ concern of contracting COVID-19 while at work and the likelihood they would maintain at least a six-foot distance from people at work, most participants who reported they were a little concerned about contracting COVID-19 at work also reported it was not at all likely they would maintain at least a six-foot distance from people at work (19.5%). About 24% of participants reported they were at least somewhat concerned/somewhat likely they would both contract COVID-19 at work and maintain at least a six-foot distance from people at work (Supplemental Table S13).

When assessing the participants’ concern of giving COVID-19 to their friends or family and the likelihood they would wear a mask inside a friend’s house, most participants reported they were a little concerned about giving COVID-19 to their friends or family and not at all likely to wear a mask while inside a friend’s house. About 17% of participants reported they were at least somewhat concerned about giving COVID-19 to their friends and family and at least somewhat likely to wear a mask inside a friend’s house.

When assessing the participants’ concern of giving COVID-19 to their friends or family and the likelihood they would maintain at least a six-foot distance from people at a gathering, most participants who reported they were a little concerned about giving COVID-19 to their family and friends also reported it was not at all likely they would maintain at least a six-foot distance from people at a gathering (18.3%). About 30% of participants reported they were at least somewhat concerned about/would be somewhat likely to both give COVID-19 to their friends and family and maintain at least a six-foot distance from people at a gathering.

## 4. Discussion

This study provides valuable insight regarding COVID-19 vaccine hesitancy among NJ teachers. The survey used in this study allowed participants to identify the different sources of information potentially influencing their decisions related to the COVID-19 safety practices and vaccines (including booster) administration. As is recent previous studies, our results indicated variation in teacher perceptions, beliefs, and attitudes towards the COVID-19 vaccines, which, thus, influenced vaccine hesitancy. A unique finding of this study was how, across the different stratifications used, U.S. governmental organizations such as the CDC and state health departments were the most trusted among teachers for relevant information related to the COVID-19 vaccines. With this study’s in-formation, public health advocates and medical professionals alike can identify the organizations to partner with in the future to challenge vaccine hesitancy and resistance efforts. Further, this study was novel since it was based on career-technical education secondary or high school teachers who supervise students and other staff and used unique demographic-based stratifications to allow us to see how different groups responded to the use of COVID-19 prevention safety methods promoted throughout the pandemic. Results help identify which demographic groups/teachers could require additional vaccine-related education efforts. By continuing to promote educational opportunities to the teachers, public health and medical professionals can continue to combat vaccine hesitancy among both teachers and the greater community. As stated in previous studies, the public health-related decisions that teachers make can influence children, adolescents, adults, and the community in general [21,22]. Further, combatting vaccine hesitancy as it relates to the COVID-19 vaccines can affect how childhood vaccination efforts are perceived in the future. Given that the COVID-19 pandemic exposed how fragile the public’s trust can be of the vaccination industry, a better understanding of the reasons behind vaccine hesitancy is necessary to address and rise above the challenges produced by emerging diseases [31]. It is important to note that the survey was provided to the teachers of interest about 12 months after the vaccine was made widely available in the U.S. With this in mind, there are various strengths and limitations to this study. One strength of this study relates to how the survey was administered to participants. The survey was released through PsychData, an online platform that was widely accessible to the population of interest. Allowing participants to complete the online survey on their own schedule and where they felt most comfortable allowed for more survey responses to be received. Another strength of this study relates to the answer options of questions provided within the survey. Multiple questions provided an answer choice labeled as “other,” where participants were given the opportunity to write in additional text they believed best represented their responses beyond the answer choices provided.

This study also had known limitations. One limitation of this study relates to the timeframe during which participants submitted their responses to the survey. Results for this set of questions might have differed if participants were asked to answer these questions at the beginning of the pandemic and/or before any vaccines were available after considering how safety protocols, such as enforced social distancing in public areas, have decreased as we have begun to enter the endemic era of COVID-19. Another limitation of this study pertains to the participants this survey was made available to. The invitation to complete this survey was released to NJ teachers who had previously completed the required courses for WBL supervision provided by NJSS through the Rutgers School of Public Health. By limiting the survey to this specific population, it is difficult to generalize our findings to different populations. Another limitation of this study relates to how all responses received were self-reported answers. Questions that asked participants to provide information such as whether they have received a positive COVID-19 diagnosis were not verified and could have questionable validity. Another limitation of this study relates to the survey’s response rate. While the target sample size was approximately 10%, it ultimately received a response rate of 23.0%. Both the target sample size and received response rate could have been higher to better verify the findings of this study.

## 5. Conclusions

The present study provides valuable insight regarding COVID-19 vaccination efforts and vaccine hesitancy trends across the state of New Jersey (NJ). When stratified by demographic variables (gender, race, and county of origin), there continued to be concern surrounding the safety and health of secondary education professionals as they continue to transition back to in-person learning and working opportunities during the on-going pandemic. Overall, most participants across gender and racial/ethnic backgrounds reported they have sought out vaccination opportunities and continue to educate themselves on the best prevention practices available to combat COVID-19 within their workplace and beyond. As survey participants resided in various counties, continuing to engage in discussions regarding vaccination efforts and additional preventative techniques could allow for better education and protection of these vulnerable populations statewide. Further, as this research study suggests, all NJ citizens, especially those residing in Cumberland or Ocean County, would benefit from a continuation in the distribution of vaccination administration and education efforts.

This study suggests recommendations to consider for future research of specific susceptible, vulnerable sub-populations. Given the largest sub-population of this study consisted of participants who identified as Non-Hispanic White, it is recommended that a more racially/ethnically diverse population is utilized to identify whether there are different trends in the responses received when compared to the original study. With most participants identifying as female in this study, it is recommended that future studies use a population consisting of nearly an equal number of females and males. By doing so, researchers will have the ability to observe if the differences in responses between females and males remains consistent or varies. Overall, additional studies should include a larger sub-population more representative of the general population, allowing researchers to draw more accurate conclusions surrounding the vaccine hesitancy related to the COVID-19 pandemic. As the COVID-19 pandemic continues to progress, educational outreach efforts will play a crucial role in convincing more citizens to receive the vaccine and limit the spread of disease, especially after considering historically low overall vaccination rates within two southern NJ counties. Future studies could also focus on whether COVID-19 vaccination rates have changed both between and within NJ counties, since this study’s survey was first administered in winter–spring 2022 as additional information about the disease was released.

## Figures and Tables

**Table 1:** Frequency counts for identified trusted sources of information about the COVID-19 vaccine overall among the NJ secondary or high school teachers based on survey responses received.

	*n* (N = 269)	% of Total	% Answered
Select your top 3 most trusted sources of information about the COVID-19 vaccine:			
Centers for Disease Control and Prevention (CDC)	176	65.4%	75.5%
Employer, family, and friends	67	24.9%	28.8%
Food and Drug Administration (FDA) ^a^	60	22.3%	25.8%
Health insurers	12	4.5%	5.2%
Hospital system websites	20	7.4%	8.6%
Local health officials	49	18.2%	21.0%
News sources	24	8.9%	10.3%
Nurses	31	11.5%	13.3%
Online publishers of medical information (such as WebMD or Mayo Clinic)	17	6.3%	7.3%
Pharmacists	24	8.9%	10.3%
Primary care providers	101	37.5%	43.3%
Professional organization(s)	9	3.3%	3.9%
Social media (such as Facebook, Twitter, Instagram, WhatsApp, LinkedIn, or TikTok)	5	1.9%	2.1%
State health departments	77	28.6%	33.0%
Other	20	7.4%	8.6%
I prefer not to answer	7	2.6%	3.0%

^a^ Prior to May 2022, the answer choices were arranged such that “Employer, family, and friends/Food and Drug Administration (FDA)“ were combined and listed as one single selection option. Following May 2022, participants were given the opportunity to select “Food and Drug Administration (FDA)” and “Employer, family and friends” separately. For this descriptive analysis, we assumed participants who completed the survey prior to this change and selected the original answer choice were counted as both “Food and Drug Administration (FDA)” and “Employer, family and friends”.

**Table 2:** Identified trusted sources of information about the COVID-19 vaccine by stratification among the NJ secondary or high school teachers. Select your top 3 most trusted sources of information about the COVID-19 vaccine.

Stratification Group ^b^		CDC ^d^	Employer, Family, Friends	FDA ^e^	Health Insurers	Hospital System Websites	Local Health Officials	News	Nurses	Online Publishers of Information	Pharmacists	Primary Care Providers	Professional Organization	Religious Leader	Social Media	State Health Departments	Other
Total (*n* = 233)	N	176	67	60	12	20	49	24	31	17	24	101	9	2	5	77	6
	%	75.5%	28.8%	25.8%	5.2%	8.6%	21.0%	10.3%	13.3%	7.3%	10.3%	43.3%	3.9%	0.9%	2.1%	33.0%	2.6%
Vaccinated (*n* = 215)	N	170	63	57	12	20	48	22	30	14	24	98	8	0	5	77	4
	%	79.1%	29.3%	26.5%	5.6%	9.3%	22.3%	10.2%	14.0%	6.5%	11.2%	45.6%	3.7%	0.0%	2.3%	35.8%	1.9%
Unvaccinated or IPNA (*n* = 18)	N	6	4	3	0	0	1	2	1	3	0	3	1	2	0	0	2
	%	33.3%	22.2%	16.7%	0.0%	0.0%	5.6%	11.1%	5.6%	16.7%	0.0%	16.7%	5.6%	11.1%	0.0%	0.0%	11.1%
Booster taken (*n* = 167) ^c^	N	137	49	4	9	15	41	17	23	11	20	83	7	0	4	58	2
	%	82.0%	29.3%	28.6%	5.4%	9.0%	24.6%	10.2%	13.8%	6.6%	12.0%	49.7%	4.2%	0.0%	2.4%	34.7%	1.2%
No booster (*n* = 48)	N	33	14	53	3	5	7	5	7	3	4	15	1	0	1	19	2
	%	68.8%	29.2%	26.4%	6.3%	10.4%	14.6%	10.4%	14.6%	6.3%	8.3%	31.3%	2.1%	0.0%	2.1%	39.6%	4.2%
“+” COVID diagnosis (*n* = 106)	N	74	27	27	5	13	22	12	14	6	8	44	3	0	1	33	3
	%	69.8%	25.5%	25.5%	4.7%	12.3%	20.8%	11.3%	13.2%	5.7%	7.5%	41.5%	2.8%	0.0%	0.9%	31.1%	2.8%
No “+” COVID diagnosis (*n* = 124)	N	99	39	32	6	6	26	12	17	11	16	55	6	0	4	44	3
	%	79.8%	31.5%	25.8%	4.8%	4.8%	21.0%	9.7%	13.7%	8.9%	12.9%	44.4%	4.8%	0.0%	3.2%	35.5%	2.4%
Female (*n* = 147)	N	118	45	44	10	12	31	19	21	9	14	65	4	0	3	51	3
	%	80.3%	30.6%	29.9%	6.8%	8.2%	21.1%	12.9%	14.3%	6.1%	9.5%	44.2%	2.7%	0.0%	2.0%	34.7%	2.0%
Male (*n* = 72)	N	52	19	14	1	8	17	3	8	5	10	32	5	0	1	23	2
	%	72.2%	26.4%	19.4%	1.4%	11.1%	23.6%	4.2%	11.1%	6.9%	13.9%	44.4%	6.9%	0.0%	1.4%	31.9%	2.8%
Non-Hispanic White (*n* = 191)	N	149	56	50	10	17	41	18	27	13	23	83	8	0	5	67	5
	%	78.0%	29.3%	26.2%	5.2%	8.9%	21.5%	9.4%	14.1%	6.8%	12.0%	43.5%	4.2%	0.0%	2.6%	35.1%	2.6%
Other (*n* = 22) ^a^	N	19	8	7	0	1	5	4	1	1	1	12	0	0	0	7	0
	%	86.4%	36.4%	31.8%	0.0%	4.5%	22.7%	18.2%	4.5%	4.5%	4.5%	54.5%	0.0%	0.0%	0.0%	31.8%	0.0%
Cumberland and Ocean counties (*n* = 24)	N	19	4	1	1	1	4	2	4	5	1	10	1	0	0	9	0
	%	79.2%	16.7%	4.2%	4.2%	4.2%	16.7%	8.3%	16.7%	20.8%	4.2%	41.7%	4.2%	0.0%	0.0%	37.5%	0.0%
Other 19 NJ counties (*n* = 205)	N	156	62	59	11	19	45	22	27	11	23	90	8	0	5	68	5
	%	76.1%	30.2%	28.8%	5.4%	9.3%	22.0%	10.7%	13.2%	5.4%	11.2%	43.9%	3.9%	0.0%	2.4%	33.2%	2.4%

Note: All answers were presented in alphabetic order. ^a^ “Other” consisted of American Indian or Alaskan Native, Middle Eastern or North African, Hispanic Asian, Hispanic Black, Hispanic White, Non-Hispanic Asian, Non-Hispanic Black, those who indicated they were multiracial, and those who self-identified as Other. ^b^ Prior to May 2022, the answer choices were arranged such that “Employer, family, and friends/Food and Drug Administration (FDA)“ were combined and listed as one single selection option. Following May 2022, participants were given the opportunity to select “Food and Drug Administration (FDA)” and “Employer, family, and friends” separately. For this descriptive analysis, we assumed participants who completed the survey prior to this change and selected the original answer choice were counted as both “Food and Drug Administration (FDA)” and “Employer, family, and friends”. ^c^ This study was not able to delineate the number of booster shots a participant had gotten at the time of taking the survey. ^d^ Center for Disease Control and Prevention (U.S.). ^e^ Food and Drug Administration (U.S.).

## Data Availability

Data were obtained via survey titled “COVID-19 Vaccine Survey for NJ teachers 2022”, which was delivered virtually to individuals who completed work-based learning (WBL) trainings provided by NJSS between 2014 and 2022.

## Supplementary Materials

The following supporting information can be downloaded at: https://www.mdpi.com/article/10.3390/vaccines11020466/s1, Table S1: Demographics of Study Sample; Table S2: COVID-19 Education Information by stratification among the NJ secondary or high school teachers.; Table S3: COVID-19 safety practice information related to social distancing by stratification among the NJ secondary or high school teachers.; Table S4: COVID-19 safety practice information related to mask wearing by stratification among the NJ secondary or high school teachers.; Table S5: Concern of contracting/infection of COVID-19 by stratification among the NJ secondary or high school teachers; Table S6: Likelihood of Wearing a Mask and Social Distancing at the Workplace (n = 230 answered both); Table S7: Likelihood of Wearing a Mask and Social Distancing in Indoor Places with Friends/Family (n = 229 answered both); Table S8: Likelihood of Wearing a Mask and Social Distancing in Public Transport (n = 231 answered both); Table S9: Likelihood of Wearing a Mask and Social Distancing in Stores (n = 232 answered both); Table S10: Level of Concern about COVID-19 and Mask Use at Workplaces (n = 231 answered both); Table S11: Level of Concern about COVID-19 and Social Distancing at Workplaces (n = 231 answered both); Table S12: Level of Concern about COVID-19 and Mask Use in Indoor Spaces with Friends/Family (n = 231 answered both); Table S13: Level of Concern about COVID-19 and Social Distancing at Social Gatherings in General (n = 230 answered both).
