# Supplementary material for: COVID-19 Vaccine Hesitancy among New Jersey Teachers and Impacts of Vaccination Information Dissemination
Source: Vaccines (Basel). 2023 Feb 17;11(2):466. doi: 10.3390/vaccines11020466 (PMC9967281; doi:10.3390/vaccines11020466)
Supplement: Supplementary file 1 [file vaccines-11-00466-s001.zip › Supplementary Material_Rutgers SPH NJSS Vaccine Hesitancy Survey 02142022.pdf]

Supplementary Material: New Jersey Safe Schools Program survey (v.2/14/2022) approved by Rutgers University/Rutgers Biomedical and Health Sciences Institutional Review Board

Vaccine Status Information

1a. Have you received a COVID-19 vaccine?

- Yes
- No→ Skip to 3.
- I prefer not to answer

1b. Did you receive a vaccine product that requires only one dose or two doses?

- One dose
- Two doses
- I don't know
- I prefer not to answer

1c. Have you received a COVID-19 booster vaccine?

- Yes
- No
- I prefer not to answer

Vaccine Confidence and Accessibility Information

2a. How safe did you think a COVID-19 vaccine was before you got the vaccine? Would you say…

- Not at all safe
- A little safe
- Moderately safe
- Very safe
- I prefer not to answer

2b. How safe do you think a COVID-19 vaccine is after you got the vaccine? Would you say…

- Not at all safe
- A little safe
- Moderately safe
- Very safe
- I prefer not to answer

3. How easy was it to get, or would it have been to get, a COVID-19 vaccine for yourself? Would you say…

- Very easy
- Somewhat easy
- Somewhat difficult
- Very difficult
- Not sure
- I prefer not to answer

4. What made it difficult, or would have made it difficult, for you to get a COVID-19 vaccine? (Select all that apply.)

- I couldn't go on my own (I have a physical limitation).
- It's too far away
- I didn't know where to go to get vaccinated.
- I didn't have transportation.
- I wasn't eligible to get a COVID-19 vaccine
- The hours of operation are inconvenient
- The waiting time was too long.
- It was difficult to find or make an appointment.
- I was too busy to get vaccinated.
- It was difficult to arrange for childcare.
- I didn't have time off work.
- Other
- Not sure
- I prefer not to answer

5. What was important for you to know to make you more confident in the COVID-19 vaccine? [Check all that apply]

- The fast production of the vaccine did not compromise its safety
- Agencies approving the vaccines are following strict rules
- My risk of getting sick with COVID-19 is bigger than the risk of side effects from the vaccine
- The vaccine cannot cause any immediate or long term injury
- It is impossible to get COVID-19 or any other disease from the vaccine itself or its components
- The vaccine works in protecting me from COVID-19
- The vaccine works in stopping the transmission of COVID-19 from one person to another
- Health agencies and WHO recommend the vaccine and agree it is safe
- I do not need any other information
- Other - please specify____________________
- I prefer not to answer

6. What else was important for you to know that made you more likely to take the COVID-19 vaccine? [Check all that apply]

- Once vaccinated I will be able to live my life with no restrictions
- Those with concerns about the vaccine have opportunities to share their opinions with the public
- Pharmaceutical companies will not make large profits from the vaccine
- Everybody will have equal access to the vaccine regardless of income or race

  There are no other reasons why so many people are sick (i.e. 5G tech., other unknown reasons)
- I will be free to choose if I get the vaccine or not with no consequences
- The school district or school that I work for required vaccination.
- I prefer not to answer

COVID Education Information

7. Do you know where to get accurate, timely information about COVID-19 vaccines?

- Yes
- No
- Not sure
- I prefer not to answer

8. Select your top 3 most trusted sources of information about COVID-19 vaccines:

- Centers for Disease Control and Prevention (CDC)
- Employer family and friends
- Food and Drug Administration (FDA)
- Health insurers
- Hospital system websites (e.g. Kaiser Permanente)
- Local health officials
- News sources (e.g., television, internet, and radio)
- Nurses
- Pharmacists
- Primary care providers
- Professional organization(s)
- Religious leader(s)
- State health departments
- Online publishers of medical information (such as WebMD or Mayo Clinic)
- Social media (such as Facebook, Twitter, Instagram, WhatsApp, LinkedIn, or TikTok)
- Union leader(s)
- Other
- I prefer not to answer

9. Do you currently have a primary care provider?

- Yes
- No
- Not Sure
- I prefer not to answer

10. Do you have any of the following conditions? [select all that apply]

- Cancer
- Immunocompromised state due to therapy or disease
- Obesity
- Diabetes (type 1 or 2)
- Cardiovascular disease
- Pulmonary disease
- Rheumatological condition
- I prefer not to answer

11. How likely are you to recommend getting the COVID-19 vaccine to others?

- Not at all likely
- Somewhat likely
- Extremely likely
- I prefer not to answer

COVID Safety Practices Information

12. How likely are you to do the following in the next 5-10 days?

- a) Maintain at least 6 feet distance from people who do not live in my home while in public spaces.
- b) Maintain at least 6 feet distance from people who do not live in my home while at small private gatherings.
- c) Maintain at least 6 feet distance from people at work.
    - ○ Not at all likely
    - ○ Somewhat likely
    - ○ Extremely likely
    - ○ I prefer not to answer

13. Suppose you had to do each of the following things in the next 5-10 days, how likely are you to wear a mask for each activity?

- a) Work in setting outside the home
- b) Use public transportation, a taxi, or a ride share service
- c) Go for a walk in your neighborhood
- d) Shop inside a store
- e) Visit inside a friend's house
- f) Visit a park or other outdoor public space
    - ○ Not at all likely
    - ○ Somewhat likely
    - ○ Extremely likely
    - ○ I prefer not to answer

14. How concerned are you of …

- a) Contracting COVID-19 at work? (For example: office and other work settings that are not your home)
- b) Contracting COVID-19 outside of work? (For example: at the grocery store, when you are using transportation, or in other aspects of your daily life)
- c) Infecting your family or friends with COVID-19?
    - Not at all concerned
    - A little concerned
    - Somewhat concerned
    - Very concerned
    - I prefer not to answer

COVID Case Information

15. Do you have any close family members (father/mother/grandfather/grandmother) older than 70 years?

- Yes, living together
- Yes, not living together
- No
- I prefer not to answer

16. Do you personally know anyone in your family, group of friends, or community networks who became seriously ill or died as a result of COVID-19?

- Yes
- No
- I prefer not to answer

17. To your knowledge, do you have or have you had COVID-19?

- Yes
- No
- I don't know
- I prefer not to answer

17a. IF "Yes," describe the level of care you received, or are receiving:

- Did not seek medical care
- Received medical care but was not hospitalized
- Was hospitalized
- I prefer not to answer

Demographic Information
18. County
19. Race/Ethnicity
20. Gender
21. Birth Year
22. Number of Years Teaching in NJ
23.Number of Years Teaching Overall
24. What is the highest education degree completed (choose from options presented)?
25. How many years of post secondary education (after high school) have you completed?

Resources:

Centers for Disease Control and Prevention. (2021). *Vaccine Confidence Survey Question Bank*. Vaccines & Immunizations. Retrieved January 20, 2022, from https://www.cdc.gov/vaccines/covid-19/vaccinate-with-confidence/rca-guide/downloads/CDC_RCA_Guide_2021_Tools_AppendixD_Surveys-508.pdf

Reno, C., Maietti, E., Fantini, M. P., Savoia, E., Manzoli, L., Montalti, M., & Gori, D. (2021). Enhancing COVID-19 Vaccines Acceptance: Results from a Survey on Vaccine Hesitancy in Northern Italy. Vaccines, 9(4), 378. MDPI AG. Retrieved from http://dx.doi.org/10.3390/vaccines9040378
