# Supplementary material for: COVID-19 Vaccine Hesitancy among New Jersey Teachers and Impacts of Vaccination Information Dissemination
Source: Vaccines (Basel). 2023 Feb 17;11(2):466. doi: 10.3390/vaccines11020466 (PMC9967281; doi:10.3390/vaccines11020466)
Supplement: Supplementary file 1 [file vaccines-11-00466-s001.zip › vaccines-2116442 sup tables 2923.pdf]

Supplemental Table 1: Demographics of Study Sample

	N (n=269)	% of total	% answered
School County region:			
North	100	37.2%	43.7%
Central	84	31.2%	36.7%
South	45	16.7%	19.7%
Missing	40	14.9%	
Race and Ethnicity:			
American Indian or Alaskan Native	1	0.4%	0.4%
Native Hawaiian or Other Asian-Pacific Islander	0	0.0%	0.0%
Middle Eastern/North African	2	0.7%	0.9%
Hispanic Asian	1	0.4%	0.4%
Hispanic Black	4	1.5%	1.7%
Hispanic White	17	6.3%	7.4%
Non-Hispanic Asian	3	1.1%	1.3%
Non-Hispanic Black	10	3.7%	4.4%
Non-Hispanic White	175	65.1%	76.4%
I prefer not to answer this question	13	4.8%	5.7%
Other	9	3.3%	3.9%
Missing	40	14.9%	
Gender			
Male	72	26.8%	31.4%
Female	148	55.0%	64.6%
Other/Multigender	3	1.1%	1.3%
I prefer not to answer	4	1.5%	1.7%
missing	40	14.9%	
**Birth Year**	1976±9.6		
Pre 1950-1959	11	4.1%	4.8%
1960-1969	49	18.2%	21.5%
1970-1979	81	30.1%	35.5%
1980-1989	70	26.0%	30.7%
1990-2000	17	6.3%	7.5%
missing	41	15.2%	
**Number of Years Teaching in NJ**	15.4±7.1		
**Number of Years of Teaching Overall**	16.2±7.4		
What is the highest education degree completed?			
Bachelor's degree	59	21.9%	25.8%
Master's degree	146	54.3%	63.8%
Doctoral degree	6	2.2%	2.6%
Other	18	6.7%	7.9%
Missing/NA/IPNA	40	14.9%	

**How many years of post-secondary education (after high school) have you completed?**	6.4±2.6		

Note: IPNA is "I prefer not to answer"

Supplemental Table 2. COVID-19 Education Information by stratification among the NJ secondary or high school teachers.

COVID-19 Vaccination Status	*Vaccinated (n=215)*	%	*Unvaccinated or IPNA (n=18)*	%	*Fisher's*	*Total*	%
Do you know where to get accurate information about the COVID-19 vaccines?							
Yes	187	87.0%	15	83.3%	0.45	202	86.7%
No	11	5.1%	2	11.1%		13	5.6%
Not sure	17	7.9%	1	5.6%		18	7.7%
Do you currently have a Primary care provider?							
Yes	197	91.6%	15	83.3%	0.28	212	91.0%
No	15	7.0%	3	16.7%		18	7.7%
Do you have any of the following conditions? [Check all that apply]							
Immunocompromised	11	5.1%	0	0.0%	1.00	11	4.7%
Obesity	31	14.4%	2	11.1%	1.00	33	14.2%
Diabetes (type 1 or 2)	10	4.7%	1	5.6%	0.60	11	4.7%
Cardiovascular disease	10	4.7%	0	0.0%	1.00	10	4.3%
Pulmonary disease	2	0.9%	1	5.6%	0.22	3	1.3%
How likely are you to recommend getting the COVID-19 vaccine?							
Not at all likely	31	14.4%	16	88.9%	<0.001***	47	20.2%
Somewhat likely	60	27.9%	1	5.6%		61	26.2%
Extremely likely	116	54.0%	0	0.0%		116	49.8%

Booster Status	*Booster taken (n=167)*	%	*No Booster (n=48)*	%	*Fisher's*	*Total*	%
Do you know where to get accurate information about the COVID-19 vaccines?							
Yes	148	88.6%	39	81.3%	0.32	187	87.0%
No	8	4.8%	3	6.3%		11	5.1%
Not sure	11	6.6%	6	12.5%		17	7.9%
Do you currently have a Primary care provider?							
Yes	151	90.4%	46	95.8%	0.72	197	91.6%
No	13	7.8%	2	4.2%		15	7.0%
Do you have any of the following conditions? [Check all that apply]							

Immunocompromised	9	5.4%	2	4.2%	1.00	11	5.1%
Obesity	24	14.4%	7	14.6%	1.00	31	14.4%
Diabetes (type 1 or 2)	10	6.0%	0	0.0%	0.12	10	4.7%
Cardiovascular disease	9	5.4%	1	2.1%	0.46	10	4.7%
How likely are you to recommend getting the COVID-19 vaccine?							
Not at all likely	12	7.2%	19	39.6%		31	14.4%
Somewhat likely	43	25.7%	17	35.4%	<0.001***	60	27.9%
Extremely likely	106	63.5%	10	20.8%		116	54.0%

COVID Diagnosis Status	*Positive diagnosis (n=106)*		*No Positive diagnosis (n=124)*		*Fisher's*	*Total*	*%*
Do you know where to get accurate information about the COVID-19 vaccines?							
Yes	89	84.0%	100	81.0%		189	82.0%
No	6	6.0%	7	6.0%	0.40	13	6.0%
Not sure	11	10.0%	7	6.0%		18	8.0%
Do you currently have a Primary care provider?							
Yes	94	89.0%	115	93.0%	0.18	209	91.0%
No	11	10.0%	7	6.0%		18	8.0%
Do you have any of the following conditions? [Check all that apply]							
Immunocompromised	4	3.8%	7	5.6%	0.55	11	4.8%
Obesity	15	14.2%	18	14.5%	1.00	33	14.3%
Diabetes (type 1 or 2)	3	2.8%	8	6.5%	0.23	11	4.8%
Cardiovascular disease	8	7.5%	2	1.6%	0.05*	10	4.3%
How likely are you to recommend getting the COVID-19 vaccine?							
Not at all likely	34	32.1%	13	10.5%		47	20.4%
Somewhat likely	26	24.5%	34	27.4%	<0.001***	60	26.1%
Extremely likely	42	39.6%	73	58.9%		115	50.0%

Gender	***Female (n=147)***	%	***Male (n=72)***	%	***Fisher's***	***Total***	%

Do you know where to get accurate information about the COVID-19 vaccines?							
Yes	128	87.1%	62	86.1%	0.37	190	86.8%
No	7	4.8%	6	8.3%		13	5.9%
Do you currently have a primary care provider?							
Yes	135	91.8%	64	88.9%	0.79	199	90.9%
No	11	7.5%	6	8.3%		17	7.8%
Do you have any of the following conditions? [Check all that apply]							
Immunocompromised	5	3.4%	5	6.9%	0.30	10	4.6%
Obesity	16	10.9%	16	22.2%	0.04*	32	14.6%
Diabetes	1	0.7%	10	13.9%	<0.001***	11	5.0%
Cardiovascular disease	7	4.8%	3	4.2%	1.00	10	4.6%
How likely are you to recommend getting the COVID-19 vaccine to others?							
Not at all likely	31	21.1%	11	15.3%	0.55	42	19.2%
Somewhat likely	36	24.5%	20	27.8%		56	25.6%
Extremely likely	74	50.3%	40	55.6%		114	52.1%

Race	***Non-Hispanic White (n=191)***	**%**	***Other (n=22)***	**%**	***Fisher's***	***Total***	**%**
Do you know where to get accurate information about the COVID-19 vaccines?							
Yes	165	86.4%	20	90.9%	1.00	185	86.9%
No	11	5.8%	1	4.5%		12	5.6%
Do you currently have a primary care provider?							
Yes	174	91.1%	21	95.5%	0.38	195	91.5%
No	16	8.4%	0	0.0%		16	7.5%
Do you have any of the following conditions? [Check all that apply]							
Immunocompromised	9	4.7%	1	4.5%	1.00	10	4.7%
Obesity	27	14.1%	5	22.7%	0.34	32	15.0%
Diabetes	11	5.8%	0	0.0%	0.61	11	5.2%
Cardiovascular disease	9	4.7%	1	4.5%	1.00	10	4.7%

How likely are you to recommend getting the COVID-19 vaccine to others?							
Not at all likely	35	18.3%	6	27.3%	0.54	41	19.2%
Somewhat likely	50	26.2%	4	18.2%		54	25.4%
Extremely likely	100	52.4%	12	54.5%		112	52.6%

County	***Cumberland and Ocean (n=24)***	***%***	***Other Counties (n=205)***	***%***	***Fisher's***	***Total***	***%***
Do you know where to get accurate information about the COVID-19 vaccines?							
Yes	20	83.3%	178	86.8%	0.63	198	86.5%
No	2	8.3%	11	5.4%		13	5.7%
Do you currently have a primary care provider?							
Yes	20	83.3%	188	91.7%	0.11	208	90.8%
No	4	16.7%	14	6.8%		18	7.9%
Do you have any of the following conditions? [Check all that apply]							
Cancer	0	0.0%	2	1.0%	1.00	2	0.9%
Immunocompromised	2	8.3%	9	4.4%	0.32	11	4.8%
Obesity	2	8.3%	31	15.1%	0.54	33	14.4%
Diabetes	2	8.3%	9	4.4%	0.32	11	4.8%
Cardiovascular disease	2	8.3%	8	3.9%	0.28	10	4.4%
Pulmonary disease	0	0.0%	3	1.5%	1.00	3	1.3%
Rheumatological condition	0	0.0%	1	0.5%	1.00	1	0.4%
How likely are you to recommend getting the COVID-19 vaccine to others?							
Not at all likely	5	20.8%	39	19.0%	0.74	44	19.2%
Somewhat likely	8	33.3%	52	25.4%		60	26.2%
Extremely likely	11	45.8%	105	51.2%		116	50.7%

Note: IPNA is “I prefer not to answer” *p=<0.05, ** p=<0.01, ***p=<0.001

Supplemental Table 3. COVID-19 safety practice information related to social distancing by stratification among the NJ secondary or high school teachers.

COVID Diagnosis Status[a]	*Positive diagnosis (n=106)*	%	*No Positive diagnosis (n=124)*	%	*Fisher's*	*Total*	%
How likely are you to maintain at least 6 feet distance from people who do not live in your home while in public spaces?							
Not at all likely	43	40.6%	53	42.7%	0.59	96	41.7%
Somewhat likely	45	42.5%	45	36.3%		90	39.1%
Extremely likely	18	17.0%	26	21.0%		44	19.1%
How likely are you to maintain at least 6 feet distance from people who do not live in your home while at small private gatherings?							
Not at all likely	64	60.4%	57	46.0%	0.12	121	52.6%
Somewhat likely	30	28.3%	44	35.5%		74	32.2%
Extremely likely	12	11.3%	21	16.9%		33	14.3%
How likely are you to maintain at least 6 feet distance from people who do not live in your home while at work?							
Not at all likely	60	56.6%	63	50.8%	0.66	123	53.5%
Somewhat likely	37	34.9%	46	37.1%		83	36.1%
Extremely likely	9	8.5%	14	11.3%		23	10.0%

COVID-19 Vaccination Status	*Vaccinated (n=215)*	%	*Unvaccinated or IPNA (n=18)*	%	*Fisher's*	*Total*	%
How likely are you to maintain at least 6 feet distance from people who do not live in your home while in public spaces?							
Not at all likely	88	40.9%	10	55.6%	0.31	98	42.1%
Somewhat likely	86	40.0%	4	22.2%		90	38.6%
Extremely likely	40	18.6%	4	22.2%		44	18.9%
How likely are you to maintain at least 6 feet distance from people who do not live in your home while at small private gatherings?							
Not at all likely	110	51.2%	12	66.7%	0.14	122	52.4%
Somewhat likely	73	34.0%	2	11.1%		75	32.2%
Extremely likely	30	14.0%	3	16.7%		33	14.2%
How likely are you to maintain at least 6 feet distance from people who do not live in your home while at work?							
Not at all likely	114	53.0%	10	55.6%	0.29	124	53.2%
Somewhat likely	80	37.2%	4	22.2%		84	36.1%
Extremely likely	20	9.3%	3	16.7%		23	9.9%
Booster Status	***Booster taken (n=167)***	%	***No Booster (n=48)***	%	*Fisher's*	*Total*	%

How likely are you to maintain at least 6 feet distance from people who do not live in your home while in public spaces?								
Not at all likely	58	34.7%	30	62.5%	0.003**	88	40.9%
Somewhat likely	75	44.9%	11	22.9%		86	40.0%
Extremely likely	33	19.8%	7	14.6%		40	18.6%
How likely are you to maintain at least 6 feet distance from people who do not live in your home while at small private gatherings?							
Not at all likely	77	46.1%	33	68.8%	0.02*	110	51.2%
Somewhat likely	64	38.3%	9	18.8%		73	34.0%
Extremely likely	24	14.4%	6	12.5%		30	14.0%
How likely are you to maintain at least 6 feet distance from people who do not live in your home while at work?							
Not at all likely	81	48.5%	33	68.8%	0.02*		53.0%
Somewhat likely	70	41.9%	10	20.8%		80	37.2%
Extremely likely	15	9.0%	5	10.4%		20	9.3%
Gender	***Female (n=147)***	*%*	***Male (n=72)***	*%*	***Fisher's***	***Total***	*%*
How likely are you to maintain at least 6 feet distance from people who do not live in your home while in public spaces?							
Not at all likely	61	41.5%	31	43.1%	0.20	92	42.0%
Somewhat likely	53	36.1%	32	44.4%		85	38.8%
Extremely likely	32	21.8%	9	12.5%		41	18.7%
How likely are you to maintain at least 6 feet distance from people who do not live in your home while at small private gatherings?							
Not at all likely	76	51.7%	39	54.2%	0.40	115	52.5%
Somewhat likely	48	32.7%	26	36.1%		74	33.8%
Extremely likely	22	15.0%	6	8.3%		28	12.8%
How likely are you to maintain at least 6 feet distance from people who do not live in your home while at work?							
Not at all likely	82	55.8%	35	48.6%	0.28	117	53.4%
Somewhat likely	49	33.3%	32	44.4%		81	37.0%
Extremely likely	15	10.2%	5	6.9%		20	9.1%
Race	***Non-Hispanic White (n=191)***	*%*	***Other (n=22)***	*%*	***Fisher's***	***Total***	*%*
How likely are you to maintain at least 6 feet distance from people who do not live in your home while in public spaces?							
Not at all likely	85	44.5%	4	18.2%	0.04*	89	41.8%

Somewhat likely	72	37.7%	11	50.0%		83	39.0%
Extremely likely	33	17.3%	7	31.8%		40	18.8%
How likely are you to maintain at least 6 feet distance from people who do not live in your home while at small private gatherings?							
Not at all likely	107	56.0%	6	27.3%	0.03*	113	53.1%
Somewhat likely	60	31.4%	12	54.5%		72	33.8%
Extremely likely	23	12.0%	4	18.2%		27	12.7%
How likely are you to maintain at least 6 feet distance from people who do not live in your home while at work?							
Not at all likely	106	55.5%	8	36.4%	0.10	114	53.5%
Somewhat likely	69	36.1%	10	45.5%		79	37.1%
Extremely likely	15	7.9%	4	18.2%		19	8.9%

County	*Cumberland and Ocean (n=24)*	%	*Other Counties (n=205)*	%	*Fisher's*	*Total*	%
How likely are you to maintain at least 6 feet distance from people who do not live in your home while in public spaces?							
Not at all likely	11	45.8%	85	41.5%	0.78	96	41.9%
Somewhat likely	10	41.7%	79	38.5%		89	38.9%
Extremely likely	3	12.5%	40	19.5%		43	18.8%
How likely are you to maintain at least 6 feet distance from people who do not live in your home while at small private gatherings?							
Not at all likely	14	58.3%	106	52.7%	0.79	120	52.4%
Somewhat likely	8	33.3%	67	32.7%		75	32.8%
Extremely likely	2	8.3%	30	14.6%		32	14.0%
How likely are you to maintain at least 6 feet distance from people who do not live in your home while at work?							
Not at all likely	9	37.5%	112	54.6%	0.08	121	52.8%
Somewhat likely	14	58.3%	70	34.1%		84	36.7%
Extremely likely	1	4.2%	22	10.7%		23	10.0%

[a]No positive diagnosis consists of both a negative diagnosis and "I do not know". I do not know means either an inconclusive test or the participant did not take a test.
Note: IPNA is "I prefer not to answer", *p=<0.05, ** p=<0.01, ***p=<0.001

Supplemental Table 4: COVID-19 safety practice information related to mask wearing by stratification among the NJ secondary or high school teachers.

COVID Diagnosis Status	*Positive diagnosis (n=106)*	*%*	*No Positive diagnosis (n=124)*	*%*	*Fisher's*	*Total*	*%*
How likely are you to wear masks while in work settings outside the home?							
Not at all likely	57	53.8%	65	52.4%		122	53.0%
Somewhat likely	17	16.0%	21	16.9%	0.95	38	16.5%
Extremely likely	31	29.2%	38	30.6%		69	30.0%
How likely are you to wear masks while using public transportation?							
Not at all likely	47	44.3%	32	25.8%		79	34.3%
Somewhat likely	22	20.8%	32	25.8%	0.01**	54	23.5%
Extremely likely	36	34.0%	60	48.4%		96	41.7%
How likely are you to wear masks while going for a walk in your neighborhood?							
Not at all likely	85	80.2%	107	86.3%		192	83.5%
Somewhat likely	7	6.6%	3	2.4%	0.25	10	4.3%
Extremely likely	14	13.2%	12	9.7%		26	11.3%
How likely are you to wear masks while shopping inside a store?							
Not at all likely	51	48.1%	51	41.1%		102	44.3%
Somewhat likely	26	24.5%	39	31.5%	0.45	65	28.3%
Extremely likely	29	27.4%	34	27.4%		63	27.4%
How likely are you to wear masks while inside a friend's house?							
Not at all likely	77	72.6%	92	74.2%		169	73.5%
Somewhat likely	16	15.1%	17	13.7%	0.95	33	14.3%
Extremely likely	12	11.3%	15	12.1%		27	11.7%
How likely are you to wear masks while in other outdoor spaces?							
Not at all likely	81	76.4%	103	83.1%		184	80.0%
Somewhat likely	9	8.5%	8	6.5%	0.54	17	7.4%
Extremely likely	15	14.2%	13	10.5%		28	12.2%

COVID-19 Vaccination Status	*Vaccinated (n=215)*	*%*	*Unvaccinated or IPNA(n=18)*	*%*	*Fisher's*	*Total*	*%*

How likely are you to wear masks while in work settings outside the home?								
Not at all likely	110	51.2%	13	72.2%	0.29	123	52.8%
Somewhat likely	36	16.7%	2	11.1%		38	16.3%
Extremely likely	67	31.2%	3	16.7%		70	30.0%
How likely are you to wear masks while using public transportation?								
Not at all likely	71	33.0%	9	50.0%	0.17	80	34.3%
Somewhat likely	49	22.8%	5	27.8%		54	23.2%
Extremely likely	93	43.3%	4	22.2%		97	41.6%
How likely are you to wear masks while going for a walk in your neighborhood?								
Not at all likely	178	82.8%	16	88.9%	1.00	194	83.3%
Somewhat likely	10	4.7%	0	0.0%		10	4.3%
Extremely likely	25	11.6%	2	11.1%		27	11.6%
How likely are you to wear masks while shopping inside a store?								
Not at all likely	90	41.9%	13	72.2%	0.06	103	44.2%
Somewhat likely	63	69.3%	3	16.7%		66	28.3%
Extremely likely	61	28.3%	2	11.1%		63	27.0%
How likely are you to wear masks while inside a friend's house?								
Not at all likely	157	73.0%	14	77.8%	1.00	171	73.4%
Somewhat likely	31	14.4%	2	11.1%		33	14.2%
Extremely likely	25	11.6%	2	11.1%		27	11.6%
How likely are you to wear masks while in other outdoor spaces?								
Not at all likely	171	79.5%	15	83.3%	1.00	186	79.8%
Somewhat likely	16	7.4%	1	5.6%		17	7.3%
Extremely likely	26	12.1%	2	11.1%		28	12.0%
Booster Status	***Booster taken (n=167)***	**%**	***No Booster (n=48)***	**%**	***Fisher's***	***Total***	**%**
How likely are you to wear masks while in work settings outside the home?								
Not at all likely	75	44.9%	35	72.9%	0.003*	110	51.2%
Somewhat likely	30	18.0%	6	12.5%		36	16.7%

Extremely likely	60	35.9%	7	14.6%		67	31.2%
How likely are you to wear masks while using public transportation?							
Not at all likely	45	26.9%	26	54.2%		71	33.0%
Somewhat likely	38	22.8%	11	22.9%	<0.001***	49	22.8%
Extremely likely	83	49.7%	10	20.8%		93	43.3%
How likely are you to wear masks while going for a walk in your neighborhood?							
Not at all likely	138	82.6%	40	83.3%		178	82.8%
Somewhat likely	8	4.8%	2	4.2%	0.94	10	4.7%
Extremely likely	19	11.4%	6	12.5%		25	11.6%
How likely are you to wear masks while shopping inside a store?							
Not at all likely	64	38.3%	26	54.2%		90	41.9%
Somewhat likely	50	29.9%	13	27.1%	0.12	63	29.3%
Extremely likely	52	31.1%	9	18.8%		61	28.4%
How likely are you to wear masks while inside a friend's house?							
Not at all likely	118	70.7%	39	81.3%		157	73.0%
Somewhat likely	28	16.8%	3	6.3%	0.19	31	14.4%
Extremely likely	19	11.4%	6	12.5%		25	11.6%
How likely are you to wear masks while in other outdoor spaces?							
Not at all likely	130	77.8%	41	85.4%		171	79.5%
Somewhat likely	16	9.6%	0	0.0%	0.05*	16	7.4%
Extremely likely	19	11.4%	7	14.6%		26	12.1%

Gender	*Female (n=147)*	%	*Male (n=72)*	%	*Fisher's*	*Total*	%
How likely are you to wear masks while in work settings outside the home?							
Not at all likely	73	49.7%	42	58.3%	0.001***	115	52.5%
Somewhat likely	17	11.6%	18	25.0%		35	16.0%

Extremely likely	55	37.4%	12	16.7%		67	30.6%
How likely are you to wear masks while using public transportation?							
Not at all likely	45	30.6%	29	40.3%		74	33.8%
Somewhat likely	30	20.4%	22	30.6%	0.02*	52	23.7%
Extremely likely	71	48.3%	21	29.2%		92	42.0%
How likely are you to wear masks while going for a walk in your neighborhood?							
Not at all likely	123	83.7%	59	81.9%		182	83.1%
Somewhat likely	7	4.8%	3	4.2%	0.75	10	4.6%
Extremely likely	15	10.2%	10	13.9%		25	11.4%
How likely are you to wear masks while shopping inside a store?							
Not at all likely	62	42.2%	33	45.8%		95	43.4%
Somewhat likely	39	26.5%	24	33.3%	0.28	63	28.8%
Extremely likely	45	30.6%	15	20.8%		60	27.4%
How likely are you to wear masks while inside a friend's house?							
Not at all likely	110	74.8%	50	69.4%		160	73.1%
Somewhat likely	22	15.0%	10	13.9%	0.25	32	14.6%
Extremely likely	13	8.8%	12	16.7%		25	11.4%
How likely are you to wear masks while in other outdoor spaces?							
Not at all likely	118	80.3%	57	79.2%		175	79.9%
Somewhat likely	13	8.8%	3	4.2%	0.33	16	7.3%
Extremely likely	15	10.2%	11	15.3%		26	11.9%

**Race[a]**	**Non-Hispanic White (n=191)**	**%**	**Other (n=22)**	**%**	**Fisher's**	**Total**	**%**
How likely are you to wear masks while in work settings outside the home?							
Not at all likely	103	53.9%	8	36.4%		111	52.1%
Somewhat likely	31	16.2%	3	13.6%	0.15	34	16.0%
Extremely likely	56	29.3%	11	50.0%		67	31.5%

How likely are you to wear masks while using public transportation?							
Not at all likely	65	34.0%	5	22.7%	0.34	70	32.9%
Somewhat likely	46	24.1%	4	18.2%		50	23.5%
Extremely likely	79	41.4%	13	59.1%		92	43.2%
How likely are you to wear masks while going for a walk in your neighborhood?							
Not at all likely	164	85.9%	14	63.6%	0.01**	178	83.6%
Somewhat likely	6	3.1%	3	13.6%		9	4.2%
Extremely likely	19	9.9%	5	22.7%		24	11.3%
How likely are you to wear masks while shopping inside a store?							
Not at all likely	85	44.5%	6	27.3%	0.02*	91	42.7%
Somewhat likely	57	29.8%	4	18.2%		61	28.6%
Extremely likely	48	25.1%	12	54.5%		60	28.2%
How likely are you to wear masks while inside a friend’s house?							
Not at all likely	145	75.9%	12	54.5%	0.03*	157	73.7%
Somewhat likely	26	13.6%	4	18.2%		30	14.1%
Extremely likely	19	9.9%	6	27.3%		25	11.7%
How likely are you to wear masks while in other outdoor spaces?							
Not at all likely	157	82.2%	14	63.6%	0.14	171	80.3%
Somewhat likely	12	6.3%	2	9.1%		14	6.6%
Extremely likely	21	11.0%	5	22.7%		26	12.2%

County	*Cumberland and Ocean (n=24)*	*%*	*Other Counties (n=205)*	*%*	*Fisher’s*	*Total*	*%*
How likely are you to wear masks while in work settings outside the home?							
Not at all likely	14	58.3%	106	51.7%	0.88	120	52.4%
Somewhat likely	3	12.5%	34	16.6%		37	16.2%
Extremely likely	7	29.2%	63	30.7%		70	30.6%
How likely are you to wear masks while using public transportation?							

Not at all likely	8	33.3%	70	34.1%	0.76	78	34.1%
Somewhat likely	7	29.2%	46	22.4%		53	23.1%
Extremely likely	9	37.5%	87	42.4%		96	41.9%
How likely are you to wear masks while going for a walk in your neighborhood?							
Not at all likely	20	83.3%	170	82.9%	0.46	190	83.0%
Somewhat likely	2	8.3%	8	3.9%		10	4.4%
Extremely likely	2	8.3%	25	12.2%		27	11.8%
How likely are you to wear masks while shopping inside a store?							
Not at all likely	12	50.0%	88	42.9%	0.76	100	43.7%
Somewhat likely	7	29.2%	58	28.3%		65	28.4%
Extremely likely	5	20.8%	58	28.3%		63	27.5%
How likely are you to wear masks while inside a friend's house?							
Not at all likely	17	70.8%	150	73.2%	0.64	167	72.9%
Somewhat likely	5	20.8%	28	13.7%		33	14.4%
Extremely likely	2	8.3%	25	12.2%		27	11.8%
How likely are you to wear masks while in other outdoor spaces?							
Not at all likely	19	79.2%	163	79.5%	0.92	182	79.5%
Somewhat likely	2	8.3%	15	7.3%		17	7.4%
Extremely likely	3	12.5%	25	12.2%		28	12.2%

Note: IPNA is "I prefer not to answer", *p=<0.05, ** p=<0.01, ***p=<0.001

ɋ "Other" consisted of: American Indian or Alaskan Native, Middle Eastern or North African, Hispanic Asian, Hispanic Black, Hispanic White, Non-Hispanic Asian, Non-Hispanic Black, those who indicated they were multiracial and those who self-identified as Other.

Supplemental Table 5: Concern of contracting/infection of COVID-19 by stratification among the NJ secondary or high school teachers.

COVID Diagnosis Status[b]	*Positive diagnosis (n=106)*	*%*	*No Positive diagnosis (n=124)*	*%*	*Fisher's*	*Total*	*%*
How concerned are you of contracting COVID-19 at work?							
Not at all concerned	35	33.0%	24	19.4%	0.10	59	25.7%

A little concerned	33	31.1%	49	39.5%		82	35.7%
Somewhat concerned	24	22.6%	28	22.6%		52	22.6%
Very concerned	14	13.2%	23	18.5%		37	16.1%
How concerned are you of contracting COVID-19 outside of work? (For example: at the grocery store, when you are using transportation, or in other aspects of your daily life)							
Not at all concerned	50	47.2%	32	25.8%	<0.001***	82	35.7%
A little concerned	28	26.4%	54	43.5%		82	35.7%
Somewhat concerned	23	21.7%	24	19.4%		47	20.4%
Very concerned	5	4.7%	14	11.3%		19	8.3%
How concerned are you of infecting your family or friends with COVID-19?							
Not at all concerned	24	22.6%	26	21.0%	0.93	50	21.7%
A little concerned	28	26.4%	32	25.8%		60	26.1%
Somewhat concerned	25	23.6%	27	21.8%		52	22.6%
Very concerned	29	27.4%	39	31.5%		68	29.6%

COVID-19 Vaccination Status	*Vaccinated (n=215)*	%	*Unvaccinated or IPNA (n=18)*	%	*Fisher's*	*Total*	%
How concerned are you of contracting COVID-19 at work?							
Not at all concerned	47	21.9%	13	72.2%	<0.001***	60	25.8%
A little concerned	80	37.2%	3	16.7%		83	35.6%
Somewhat concerned	53	24.7%	0	0.0%		53	22.7%
Very concerned	35	16.3%	2	11.1%		37	15.9%
How concerned are you of contracting COVID-19 outside of work? (For example: at the grocery store, when you are using transportation, or in other aspects of your daily life)							
Not at all concerned	72	33.5%	13	72.2%	0.02*	85	36.5%
A little concerned	79	36.7%	3	16.7%		82	35.2%
Somewhat concerned	46	21.4%	1	5.6%		47	20.2%
Very concerned	18	8.4%	1	5.6%		19	8.2%
How concerned are you of infecting your family or friends with COVID-19?							

Not at all concerned	40	18.6%	12	66.7%	<0.001***	52	22.3%
A little concerned	59	27.4%	2	11.1%		61	26.2%
Somewhat concerned	50	23.3%	2	11.1%		52	22.3%
Very concerned	66	30.7%	2	11.1%		68	29.2%

Booster Status	*Booster taken (n=167)*	%	*No Booster (n=48)*	%	*Fisher's*	*Total*	%
How concerned are you of contracting COVID-19 at work?							
Not at all concerned	28	16.8%	19	39.6%	0.02*	47	21.9%
A little concerned	67	40.1%	13	27.1%		80	37.2%
Somewhat concerned	43	25.7%	10	20.8%		53	24.7%
Very concerned	29	17.4%	6	12.5%		35	16.3%
How concerned are you of contracting COVID-19 outside of work? (For example: at the grocery store, when you are using transportation, or in other aspects of your daily life)							
Not at all concerned	47	28.1%	25	52.1%	0.01**	72	33.5%
A little concerned	63	37.7%	16	33.3%		79	36.7%
Somewhat concerned	41	24.6%	5	10.4%		46	21.4%
Very concerned	16	9.6%	2	4.2%		18	8.4%
How concerned are you of infecting your family or friends with COVID-19?							
Not at all concerned	28	16.8%	12	25.0%	0.60	40	18.6%
A little concerned	46	27.5%	13	27.1%		59	27.4%
Somewhat concerned	41	24.6%	9	18.8%		50	23.3%
Very concerned	52	31.1%	14	29.2%		66	30.7%

Gender	*Female (n=147)*	%	*Male (n=72)*	%	*Fisher's*	*Total*	%
How concerned are you of contracting COVID-19 at work?							
Not at all concerned	32	21.8%	23	31.9%	0.10	55	25.1%
A little concerned	48	32.7%	28	38.9%		76	34.7%
Somewhat concerned	38	25.9%	14	19.4%		52	23.7%

Very Concerned	29	19.7%	7	9.7%		36	16.4%
How concerned are you of contracting COVID-19 outside of work? (For example: at the grocery store, when you are using transportation, or in other aspects of your daily life)							
Not at all concerned	47	32.0%	32	44.4%	0.08	79	36.1%
A little concerned	50	34.0%	26	36.1%		76	34.7%
Somewhat concerned	35	23.8%	12	16.7%		47	21.5%
Very Concerned	15	10.2%	2	2.8%		17	7.8%
How concerned are you of infecting your family or friends with COVID-19?							
Not at all concerned	26	17.7%	21	29.2%	0.09	47	21.5%
A little concerned	38	25.9%	22	30.6%		60	27.4%
Somewhat concerned	32	21.8%	14	19.4%		46	21.0%
Very Concerned	51	34.7%	15	20.8%		66	30.1%

Race[b]	***Non-Hispanic White (n=191)***	*%*	***Other (n=22)***	*%*	***Fisher's***	***Total***	*%*
How concerned are you of contracting COVID-19 at work?							
Not at all concerned	50	26.2%	3	13.6%	0.002**	53	24.9%
A little concerned	67	35.1%	9	40.9%		76	35.7%
Somewhat concerned	50	26.2%	1	4.5%		51	23.9%
Very Concerned	24	12.6%	9	40.9%		33	15.5%
How concerned are you of contracting COVID-19 outside of work? (For example: at the grocery store, when you are using transportation, or in other aspects of your daily life)							
Not at all concerned	72	37.7%	4	18.2%	0.08	76	35.7%
A little concerned	67	35.1%	9	40.9%		76	35.7%
Somewhat concerned	41	21.5%	5	22.7%		46	21.6%
Very Concerned	11	5.8%	4	18.2%		15	7.0%
How concerned are you of infecting your family or friends with COVID-19?							
Not at all concerned	42	22.0%	3	13.6%	0.67	45	21.1%
A little concerned	54	28.3%	6	27.3%		60	28.2%

Somewhat concerned	41	21.5%	4	18.2%		45	21.1%
Very Concerned	54	28.3%	9	40.9%		63	29.6%

County	*Cumberland and Ocean (n=24)*	%	*Other Counties (n=205)*	%	*Fisher's*	*Total*	%
How concerned are you of…							
Contracting COVID-19 at work?							
Not at all concerned	6	25.0%	51	24.9%	0.16	57	24.9%
A little concerned	13	54.2%	69	33.7%		82	35.8%
Somewhat concerned	4	16.7%	49	23.9%		53	23.1%
Very Concerned	1	4.2%	36	17.6%		37	16.2%
Contacting COVID-19 outside of work? (For example: at the grocery store, when you are using transportation, or in other aspects of your daily life)							
Not at all concerned	9	37.5%	74	36.1%	0.51	83	36.2%
A little concerned	9	37.5%	71	34.6%		80	34.9%
Somewhat concerned	6	25.0%	41	20.0%		47	20.5%
Very Concerned	0	0.0%	19	9.3%		19	8.3%
Infecting your family or friends with COVID-19?							
Not at all concerned	6	25.0%	43	21.0%	0.20	49	21.4%
A little concerned	9	37.5%	52	25.4%		61	26.6%
Somewhat concerned	6	25.0%	45	22.0%		51	22.3%
Very concerned	3	12.5%	65	31.7%		68	29.7%

Note: IPNA is "I prefer not to answer", *p=<0.05, ** p=<0.01, ***p=<0.001

[a]No positive diagnosis consists of both a negative diagnosis and "I do not know". I do not know means either an inconclusive test or the participant did not take a test.

[b]"Other" consisted of: American Indian or Alaskan Native, Middle Eastern or North African, Hispanic Asian, Hispanic Black, Hispanic White, Non-Hispanic Asian, Non-Hispanic Black, those who indicated they were multiracial and those who self-identified as Other.

Supplemental Table 6: Likelihood of Wearing a Mask and Social Distancing at the Workplace (n=230 answered both)

		How likely are you to maintain at least 6 feet distance from people at work?						
		Not at all likely	%	Somewhat likely	%	Extremely likely	%	P-Value
How likely are you to wear a mask in a work setting outside the home?	Not at all likely	87	37.8%	30	13.0%	6	2.6%	<0.001***
	Somewhat likely	11	4.8%	20	8.7%	6	2.6%	
	Extremely likely	26	11.3%	33	14.4%	11	4.8%	

Note: Percentage is percentage of total table count. *p=<0.05, ** p=<0.01, ***p=<0.001

Most participants who reported it was not at all likely they would wear a mask while in a work setting outside the home also reported it was not at all likely they would maintain at least a 6 feet distance from people at work (87, 37.8%).

About 5% of participants who reported it was extremely likely they would wear a mask in a work setting outside the home also reported it was extremely likely they would maintain at least a 6 feet distance from people at work (n=11).

About 14% of participants who reported it was extremely likely they would wear a mask in a work setting outside the home also reported it was somewhat likely they would maintain at least a 6 feet distance from people at work (n=33)

Supplemental Table 7: Likelihood of Wearing a Mask and Social Distancing in Indoor Places with Friends/Family (n=229 answered both)

		How likely are you to maintain at least 6 feet distance from people at small gatherings?						
		Not at all likely	%	Somewhat likely	%	Extremely likely	%	P-Value
How likely are you to wear a mask inside a friend's house?	Not at all likely	100	43.7%	50	21.8%	19	8.3%	0.003**
	Somewhat likely	8	3.5%	15	6.6%	10	4.4%	
	Extremely likely	14	6.1%	9	3.9%	4	1.8%	

Note: Percentage is percentage of total table count. *p=<0.05, ** p=<0.01, ***p=<0.001

Supplemental Table 8: Likelihood of Wearing a Mask and Social Distancing in Public Transport (n=231 answered both)

		How likely are you to maintain at least 6 feet distance from people who do not live in your home?						
How likely are you to wear a mask while on public transportation?		Not at all likely	%	Somewhat likely	%	Extremely likely	%	P-Value
	Not at all likely	49	21.2%	25	10.8%	6	2.6%	<0.001***
	Somewhat likely	26	11.3%	21	9.1%	7	3.0%	
	Extremely likely	23	10.0%	43	18.6%	31	13.4%	

Note: Percentage is percentage of total table count. *p=<0.05, ** p=<0.01, ***p=<0.001

Most participants who reported it was not at all likely they would wear a mask while on public transportation also reported it was not at all likely they would maintain at least a 6 feet distance from people who do not live in your home (49, 21.2%).

About 44% of participants reported it would be at least somewhat likely they would both wear a mask while on public transportation and maintain at least a 6 feet distance from people who do not live in your home (n=102).

Supplemental Table 9: Likelihood of Wearing a Mask and Social Distancing in Stores (n=232 answered both)

		How likely are you to maintain at least 6 feet distance from people who do not live in your home?						
How likely are you to wear a mask while at a store?		Not at all likely	%	Somewhat likely	%	Extremely likely	%	P-Value
	Not at all likely	69	29.7%	26	11.2%	8	3.5%	<0.001***
	Somewhat likely	15	6.5%	38	16.4%	13	5.6%	
	Extremely likely	14	6.0%	26	11.2%	23	9.9%	

Note: Percentage is percentage of total table count. *p=<0.05, ** p=<0.01, ***p=<0.001

Most participants who reported it was not at all likely they would wear a mask while at a store also reported it was not at all likely they would maintain at least a 6 feet distance from people who do not live in your home (69. 29.7%).

About 43% of participants reported it would be at least somewhat likely they would both wear a mask while at a store and maintain at least a 6 feet distance from people who do not live in your home (n=100).

Supplemental Table 10: Level of Concern about COVID-19 and Mask Use at Workplaces (n=231 answered both)

	How likely are you to wear a mask in a work setting outside the home?							
		Not at all likely	%	Somewhat likely	%	Extremely likely	%	P-Value
How concerned are you about contracting COVID-19 at work?	Not at all concerned	51	22.1%	3	1.3%	5	2.2%	<0.001***
	A little concerned	44	19.0%	18	7.8%	21	9.1%	
	Somewhat concerned	19	8.2%	12	5.2%	21	9.1%	
	Very concerned	9	3.9%	5	2.2%	23	10.0%	

Note: Percentage is percentage of total table count. *p=<0.05, ** p=<0.01, ***p=<0.001

Most participants who reported they were not at all concerned about contracting COVID-19 at work also reported it was not at all likely they would wear a mask in a work setting outside the home (51, 22.1%).

About 27% of participants reported they were at least somewhat concerned/would be somewhat likely they would both contract COVID-19 at work and wear a mask in a work setting outside the home.

Supplemental Table 11: Level of Concern about COVID-19 and Social Distancing at Workplaces (n=231 answered both)

	How likely are you to maintain at least 6 feet distance from people at work?							
		Not at all likely	%	Somewhat likely	%	Extremely likely	%	P-Value
How concerned are you about contracting COVID-19 at work?	Not at all concerned	43	18.7%	11	4.8%	4	1.7%	<0.001***
	A little concerned	45	19.5%	33	14.3%	5	2.2%	
	Somewhat concerned	22	9.5%	26	11.3%	5	2.2%	
	Very concerned	14	6.1%	14	6.1%	9	3.9%	

Percentage is percentage of total table count. *p=<0.05, ** p=<0.01, ***p=<0.001

Most participants who reported they were a little concerned about contracting COVID-19 at work also reported it was not at all likely they would maintain at least a 6 feet distance from people at work (45, 19.5%).

About 24% of participants reported they were at least somewhat concerned/somewhat likely they would both contract COVID-18 at work and maintain at least a 6 feet distance from people at work (n=54).

Supplemental Table 12: Level of Concern about COVID-19 and Mask Use in Indoor Spaces with Friends/Family (n=231 answered both)

	How likely are you to wear a mask inside a friend's house?							
		Not at all likely	%	Somewhat likely	%	Extremely likely	%	P-Value
How concerned are you about giving COVID-19 to your friends or family?	Not at all concerned	42	18.2%	3	1.3%	6	2.6%	0.02*
	A little concerned	50	21.7%	5	2.2%	6	2.6%	
	Somewhat concerned	38	16.5%	6	2.6%	7	3.0%	
	Very concerned	41	17.8%	19	8.2%	8	3.5%	

Percentage is percentage of total table count. *p=<0.05, ** p=<0.01, ***p=<0.001

Most participants who reported they were a little concerned about giving COVID-19 to their friends or family and not at all likely to wear a mask while inside a friend's house.

About 17% of participants reported they were at least somewhat concerned about giving COVID-19 to their friends and family and at least somewhat likely to wear a mask inside a friend's house (n=40)

Supplemental Table 13: Level of Concern about COVID-19 and Social Distancing at Social Gatherings in General (n=230 answered both)

	How likely are you to maintain at least 6 feet distance from people at a gathering?							
		Not at all likely	%	Somewhat likely	%	Extremely likely	%	P-Value
How concerned are you about giving COVID-19 to your friends or family?	Not at all concerned	36	15.7%	9	3.9%	4	1.7%	<0.001***
	A little concerned	42	18.3%	14	6.1%	5	2.2%	
	Somewhat concerned	24	10.4%	21	9.1%	7	3.0%	
	Very concerned	20	8.7%	31	13.5%	17	7.4%	

Percentage is percentage of total table count. *p=<0.05, ** p=<0.01, ***p=<0.001

Most participants who reported they were a little concerned about giving COVID-19 to their family and friends also reported it was not at all likely they would maintain at least a 6 feet distance from people at a gathering (42, 18,3%)

About 30% of participants reported they were at least somewhat concerned/would be somewhat likely about both giving COVID-19 to their friends and family and maintain at least a 6 feet distance from people at a gathering (n=76).
